# Remote interpreting in primary care settings: a feasibility trial in Germany

**DOI:** 10.1186/s12913-021-07372-6

**Published:** 2022-01-24

**Authors:** Jonas Fiedler, Susanne Pruskil, Christian Wiessner, Thomas Zimmermann, Martin Scherer

**Affiliations:** 1 Department of General Practice / Primary Care, University Medical Center Hamburg-Eppendorf, Hamburg, Germany; 2 Public health department, Altona, Hamburg, Germany; 3 Centre for Experimental Medicine, Department of Biometry and Epidemiology, University Medical Centre Hamburg-Eppendorf, Hamburg, Germany

**Keywords:** Migration, Patient centeredness, Language discordant patients, Healthcare, Primary care, Video remote interpreting, Telephone remote interpreting

## Abstract

**Background:**

Global migration trends have led to a more diverse population in health care services everywhere, which in turn has set off a paradigm shift away from medical paternalism toward more patient autonomy. Consequently, physicians need to provide a more precise patient-centred healthcare. Professional interpreting appears to play a crucial part in tackling the challenges of language barriers adequately. The aim of this study was to conduct process evaluation through the implementing of video remote interpreting (VR) and telephone remote interpreting (TR) within primary care facilities in the northern German metropolis of Hamburg.

**Methods:**

We conducted a three-armed exploratory pilot trial, which compared VR to TR and to a control group (CG) in different primary care settings. We assessed feasibility of implementation, as well as the acceptance of interpreting tools among their users. In addition, we compared the quality of communication as perceived by patients and physicians, as well as the enabling of patient-centred medicine over all three study groups using quantitative questionnaires.

**Results:**

13 practices (7 GPs, 3 Gynaecologists, 3 Paediatricians) took part in this trial. 183 interpreting calls were documented, 178 physicians as well as 127 patients answered their respective questionnaires. The implementation of the VR- und TR-tools went smoothly and they were broadly accepted by their users. However, the tools were used significantly less often than we had anticipated. With regards to quantitative questionnaires, VR scored significantly better than the control group in terms of the perceived quality of communication by both, patients and physicians and enabled of patient-centred medicine.

**Conclusion:**

Our main findings were the discrepancy between the assumed high demand of professional interpreting solutions on the one hand and the low willingness of practices to participate on the other. The rather low utilisation rates were also noteworthy. This discrepancy indicates a lack of awareness concerning the adverse effects of using informal or no interpreter in medical settings, which needs to be rectified. Due to the small sample size, all statistical results must be viewed with caution. However, our results show that remote interpreting represents a promising approach to tackling language barriers in primary care settings.

**Supplementary Information:**

The online version contains supplementary material available at 10.1186/s12913-021-07372-6.

## Background

Over the course of the past decades, two major developments in western medicine are notable: mass migration on a global scale and a simultaneous shift towards individualised patient-centred medicine. Global migration has increased during this period resulting in more diverse populations than ever before. During this period of recent history, Germany welcomed the second largest number of migrants, second only to the United States [1]. Consequently, there are more than twenty million people with a migratory background living in Germany according to the German Census Bureau [2]. It is thus important to address the topic of healthcare provision for this increasingly large group of the German population. Schouten et al. identify the second development as “a paradigm shift away from medical paternalism toward more patient autonomy” (Schouten et al., 2009, p. 469). Patient centred medicine has shown to provide better health outcomes and improve patient satisfaction [3, 4].

Modern healthcare professionals face the challenge of providing patient-centred medicine to an increasingly diverse patient population. As early as 1997, Crane found that patients considered their doctors to be the most important source of medical information and that doctor-patient communication was impeded by language discordance [5]. Meanwhile, accurate medical history taking is crucial in every diagnostic process [6]. Language barriers therefore constitute one of the most significant barriers to the provision of adequate healthcare for this diverse group of patients [7, 8].

The reliance on informal interpreters, such as family members or medical staff is considered to be mostly insufficient and inadequate. Indeed, informal interpreters provided less accurate interpreting [9]. Furthermore, the reliance on ad hoc interpreters is one of the three main causes of errors in the treatment of limited English proficiency (LEP) patients [10]. Finally, errors committed by ad hoc interpreters potentially lead to more severe consequences than errors committed by professional interpreters [11, 12]. In contrast to these findings, studies have shown that doctors felt the healthcare they provided improved with the use of professional interpreters [13]. A systematic review by Karliner et al. showed that patients understood their diagnosis better with professional interpreting, utilisation of medical services equalled those of language-concordant patients, and clinical outcomes as well as satisfaction rates were also both higher with than without professional interpreting as opposed to without [14]. Yet, informal interpreters are still relied on in the vast majority of the cases [15–17].

The most important arguments against the use of professional interpreters, such as their insufficient availability, high costs [18] and time constraints [19] can be overcome by relying on remote interpreting services [20]. They have been recommended for their “instant and 24-hour availability” (Leman, 1997, p. 98). The amount of time required for remote interpreting equalled the time needed for in-person interpreting [21], thus reducing the interpreting costs [22]. Video remote interpreting (VR) proved to “increase the quality of the conversation with the patient “(Mottelson et al., 2018, p. 246). Clinicians rated VR as equally beneficial as in-person interpreting and significantly better than ad hoc interpreting [23] and, in one study, telephone remote interpreting (TR) was not rated inferior to the gold standard presented by bilingual physicians [24].

All studies included in the systematic review comparing telephone or video remote interpreting with in-person interpreting– as well as almost all the studies cited above – were conducted in hospital settings [25]. Primary care settings differ significantly for several reasons, particularly regarding language discordance and interpreting. General practitioners are often the first doctors to attend to a patient, which makes good communication particularly important. Additionally, patients often show up unannounced at primary care facilities so that the providing adequate interpreting represents a challenge. Lastly, primary care facilities, unlike hospital, usually do not have interpreting pools at their disposal.

Remote interpreting tools have already been used in practice in Hamburg. *SAVD Videodolmetschen has* provided VR for the purpose of facilitating communication between patients and doctors throughout medical care in refugee first reception centres in Hamburg [26]. Many of these patients receive regular healthcare services now, despite the language barrier still applying to the majority of this group. The lack of professional interpreting in primary care settings therefore continues to be the main challenge for an adequate provision of healthcare services to this group of patients [27]. However, there are no large-scale studies comparing TR and VR with one another and with control groups in primary care settings in the German-speaking areas. Therefore, knowledge about the implementation, usage, and evaluation of such means of interpreting is still lacking.

Seeing as the large-scale introduction of remote interpreter services into daily routines is expensive and requires a lot of resources, we conducted this feasibility trial. In order to prepare for a large-scale study, the aim of this pilot study was thus to conduct a process evaluation of the implementation of VR and TR. Such professional interpreting solutions were implemented and utilised to overcome existing language barriers in primary care settings in the northern metropolis of Hamburg.

## Methods and material

We conducted a three-pronged exploratory pilot trial comparing VR and TR with each other and with a control group (CG) and assessed the implementation feasibility as well as the acceptance of the interpreting tools among their users. Using quantitative questionnaires, we compared the perceived quality of communication and the enablement of patient-centred medicine of all three study groups. The results of the focus groups and interviews that we had additionally conducted at the end of the six-month data collection period are still unpublished data.

**Fig. 1:**
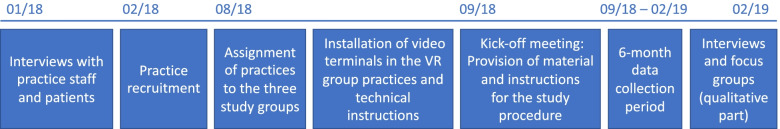
Sequence of study

Based on the experience from a similar study conducted earlier in Vienna, Austria [28], we included medical practice staff as well as patients early in the conceptual design of the study. In line with this recommendation, we conducted interviews with representatives of all three medical practice types and two interviews with language discordant patients who needed translations throughout medical consultations. Figure 1 shows the sequential process of our project.

### Participants

#### Practice recruitment

Practice recruitment followed three principles: Professional interpreting resources should be accessible (1) all over the federal state of and the city of Hamburg, (2) in areas with a high demand of professional interpreting and (3) at low-threshold medical settings. The sample of primary care practices was drawn from a publicly available list of all doctors belonging to the Association of Statutory Health Insurance Physicians [29]. Pursuant to the three practice recruitment principles, we took two neighborhoods from every district that had the highest ratio of people with a migratory background based on the data provided by the statistical office for Hamburg and Schleswig-Holstein [30].

Technical knowledge, computational, and financial restrictions limited possible participating practices to a feasible number in this explorative trial. Thus, for our pragmatic sample of practices we wanted to engage primary care practices in pediatric care: 3, in obstetrics and gynecology: 3, and general medicine: 9. In a first wave, all available practices in the neighborhoods as stratified above were sent a letter which included a description of the study as well as a declaration of consent the physicians were asked to send back by fax in case, they were interested in participating in the study.

99% practices did not respond (see Table 1). All of them (*N* = 591) were called in a second wave of recruitment, and then sent the invitation again via fax. As response remained still low, we extended recruitment into a third wave: practices that cared for refugees in the refugee first reception centres (https://youtu.be/qswTW3fTcPA), teaching practices of the Hamburg University Medical Center, Department of General Practice, as well as personal contacts.

**Table 1:** Practice recruitment process

				contacted			consented	
Phase	Timeline	Contact	General medicine	Obstetrics & Gynaecology	Paediatrics	General medicine	Obstetrics & Gynaecology	Paediatrics
1	0	letter	593	24	17	**2**	0	0
	4 weeks	phone call	591	24	17	**1**	**1**	**1**
	4 weeks	fax	491	19	2	**1**	0	0
2	10 weeks	fax	29	12	33	**3**	0	**1**
	14 weeks	phone	26	12	32	0	**2**	**1**

Overall, practice recruitment took unexpected 6 months. Eventually, we managed to include 7 general medicine practices, 3 gynecological practices and 3 pediatric practices.

All primary care practices were randomly assigned to one of the three study groups by the drawing of lots as shown in Table 2.

**Table 2:** Distribution of primary care practices among the three study groups

	VR	TR	CG
General practitioners	3	3	1
Obstetricians/Gynaecologists	1	1	1
Paediatricians	1	1	1

### Patient recruitment

We used convenience sampling. Every patient entering the medical practice was evaluated for inclusion and exclusion criteria, based on the perception of a potential language barrier as identified by on site medical staff. Inclusion criteria consisted of insufficient German language skills but native speaker proficiency in one of the following languages: Arabic, Farsi, Dari, Kurdish (Badinani), Russian, Turkish and Tigrinya. These languages were selected as they constitute the five most common languages used in first-reception centres between the years 2015–2019. Turkish was included because the Turkish population constitutes the largest group of migrants in Hamburg. Tigrinya was also added as it is the most widely spoken formal African language. Exclusion criteria were illiteracy, cognitive impairment hinting at the potential missing capacity to give informed consent (e.g. dementia) and a medical emergencies. The interpreting tool were made available to all patients, although patients fulfilling one or more of the exclusion criteria could not be recruited. Those patients who met the inclusion criteria were asked in which language they preferred to be seen by the doctor. A chart displaying different languages, as displayed in Additional file 1, was used in order to establish linguistic common ground for those rare cases where initially no form of verbal communication seemed possible. If their language of choice was one of the above, they were handed an information sheet and a declaration of informed consent. Participation in the study was voluntary and non-participation had no negative consequences. As the medical practices had to conduct the data collection during their daily routine, they were only able to properly document the included patients. Therefore, we are not able to provide data covering all the possible participants who were approached during the six-month data collection period.

### Interventions

Once the patients had given their informed consent, they attended the regular consultation. The only difference – depending on the study group – was that a professional interpreter was introduced to the consultation via telephone or video. VR tools as well as the physicians’ telephones were registered beforehand and thus ready to be relied on during the consultations. The physician and patient of the VR group, both faced the VR tool. The desired language could be selected on a touchscreen and a professional interpreter appeared on the screen ready for interpreting within 120 s. Physicians of the TR group dialled the number of the interpreting service plus a few extra digits for the language required. Just as in the VR group, an interpreter was ready to interpret within 120 s. Patients whose first language was not included in the study but still needed interpreting services could also use the interpreting tools. Their calls were excluded from call analyses later on and they did not fill out any questionnaires.

### Outcomes

#### Call analyses

Over the course of the six-month study period, every telephone call made with the help of an interpreting tool was automatically documented. The recorded data included date, time, and duration of each call as well as the language spoken and the medical practice making the call. The calls and videos were not recorded.

### Questionnaires

The questionnaires aimed to assess the perceived quality of communication of both patients and doctors as well as the degree to which patient-centred medicine could be improved through the support of professional interpreting. Both intervention groups also filled out a questionnaire examining their acceptance of the tool used during the consultation. In cases of paediatric consultations, parents/custodians filled in the questionnaires. Table 3 gives an overview of the questionnaires used.

**Table 3:** Overview of questionnaires

Name	Reference	Study group	Number of items	Rating scale
*Acceptance of the interpreting tools*	[31]	TR, VR	5	6
*Perceived quality of communication (PQC)*	[32]	CG, TR, VR	8	6
*Patient enablement index (PEI)*	[33]	CG, TR, VR	6	4
*Fragebogen zur partizipativen Entscheidungsfindung [Participative decision making questionnaire] (PEF-FB-9)*	[34]	CG, TR, VR	9	6

### Acceptance of interpreting tools

Following each consultation, the doctors and patients of the intervention groups evaluated their acceptance of the interpreting tools. We adapted and translated a questionnaire, developed by Langer and Wirth a study evaluating telephone remote interpreting services [31]. The questionnaire covered organisational aspects of the tool and assessed whether users considered further use of the interpreting tool to be beneficial. Study participants had to state to what extent (1 = *applies not at all* to 6 = *completely applies)* they agreed with each of the following five statements: the tool facilitated the communication with the doctor/patient; the tool helped me to better present my issue/understand the patient; the tool helped me/the patient to better understand the doctor’s/my questions and explanations; the tool was user-friendly, I/the patient would benefit from further use of the tool in the future. All questions were equally weighted, and a sum score was calculated. Primary outcome was the acceptance of the tool among its users.

### Patient-centred medicine

In order to examine to what extent, the tested methods of interpreting enabled patient-centred medicine, we used two validated questionnaires.

First we wanted to test the influence of professional interpreting on patient enablement “which is related to but different from general satisfaction” as Howie et al. pointed out (Howie et al., 1998, p. 165). Consequently, we decided to use the *Patient Enablement Index* (PEI).

Additionally, we included the *Fragebogen zur Partizipativen Entscheidungsfindung* (PEF-FB-9) [34] – the German version of the 9-item Shared Decision-Making Questionnaire (SDM-Q-9). The PEF-FB-9 assessed to what extent a patient had been included into a shared decision-making process [35].

All questionnaire scores were calculated in accordance with their creator’s instructions, as had been they applied in their original studies. If no suggestions were provided, we attempted to adapt the score building principles to the ones we had previously found. Accordingly, missing values were replaced by the mean value if a maximum of one (PQC, PEI and the acceptance of interpreting tools) value was missing (or two for the PEF-FB-9). Subsequently, all values were transformed to range from 0 (low) to 100 (high) for better comparability.

All questionnaires were translated into the seven languages included in this study and translated back into the original language afterwards.

Data quality control was ensured by setting ranges for data values of different items during the data entry phase. Prior to any analysis of the data collected, each questionnaire was visually inspected and consequently checked for inconsistencies.

### Perceived quality of communication

The questionnaire assessing the patients’ and the physicians’ perceived quality of communication was built on the communication model by Bird and Cohen-Cole. The authors’ aim was to develop a model that “gives equal prominence to informational, psychological and educational aspects of the interview. Furthermore, the model emphasizes pragmatic, goal-oriented and instrumental aspects of physicians’ interviewing behaviour” (Bird and Cohen-Cole, 1990, p. 69). As we considered this model to be both, global and concise, we included the authors’ three dimensions “collecting information”, “responding to the patient’s emotions” and “educating the patient” to this questionnaire.

### Data analysis

The questionnaires were collected by JF. Data entry was conducted using EpiData.

IBM SPSS Statistics 25 was used to analyse the questionnaire data obtained. Normality of the data was assessed by visual inspection of the histograms for the different scores. As the scores were not normally distributed, we carried out the non-parametric Kruskal-Wallis Test. Afterwards, a Dunn-Bonferroni test was conducted as a post hoc test in order to determine which of the groups significantly differed from the others. Additionally, a subgroup analysis was conducted comparing the VR group to the control group in the paediatric care setting using Mann-Whitney tests. Descriptive analyses were conducted for the questionnaire by Langer and Wirth. The information gathered during the telephone calls made throughout the study enrolment was processed, visualised and prepared using Microsoft Excel (Microsoft Office 365 ProPlus).

### *SAVD Videodolmetschen GmbH* – provider of the interpreting services

*SAVD Videodolmetschen GmbH* was founded in March 2014 as a result of the task force *Dealing with non-German-speaking patients* which was founded by the *Austrian Network for Patient Safety.* Every interpreter working for SAVD has completed either a master’s degree in translation studies, a corresponding qualification if such a master’s degree was not available for their language or a judicial certification. All interpreters are subject to confidentiality [36].

### CISCO Webex DX80

Please refer to the data sheet provided by Cisco [37] or contact the authors for detailed information on the video terminals used in this study.

## Results

### Feasibility

Video and telephone remote interpreting was applied in doctor-patient consultations between September 2018 and February 2019. The technical installation went straightforward for both groups and the technical support was contacted only occasionally during the data collection period.

The call analyses recorded by *SAVD Videodolmetschen GmbH* verified a total of 202 calls during the 26 weeks of enrolment. 19 video calls were excluded due to using languages not included in this study (e.g. Albanian) leaving 183 calls for further analyses. Arabic was the most commonly requested language (66 calls), followed by Farsi (39) and Dari (29). Calls lasted 14 min on average. There were no significant differences regarding the duration of calls between the three different medical specialties.

### Acceptance of interpreting tools

The overall acceptance of the video and telephone interpreting tools was very high. In response to each of the five questions, over 90% of the patients as well as the physicians stated that they either completely or mostly agreed that the interpreting tools helped both sides to be more satisfied with the consultation*.* A detailed overview of the responses is shown in the corresponding tables in Additional file 2.

### Questionnaire scores

We observed that the number of the completed questionnaires varied between the two groups. Almost all physicians filled out the questionnaire after the appointment whereas the patients only did so in about two-thirds of the cases. We attribute this discrepancy to the everyday levels of commotion in medical practices. The on site staff was asked to ensure all patients fill in the questionnaires while they were still physically in the practice, but due to busy schedules and other activities this may have not always been possible. In the following, patient questionnaire results (*n* = 127 out of the 183 calls) will be presented first, followed by the physician questionnaire results (*n* = 178 out of the 183 calls).

### Patient questionnaires

#### Sociodemographic data of the patients

Over the course of the study period 127 questionnaires (69%) were filled in and returned by the patients. A detailed description of the study population is shown in Table 4. Seventy-three participants belonged to the VR group, 14 to the TR group and 40 to the control group. 50% of the participants were female, 37% were male, 6% did not wished not to specify their gender and 7% did not provide any information. Patients belonging to the control group were younger and more often male. The patients’ educational and professional qualifications were higher in the TR group. The most common language among the returned questionnaires was Arabic making up approximately 45% of the forms, followed by Farsi (19%) and Tigrinya (13%). 61% of the consultations took place in paediatric, 24% in obstetrics and gynaecology and 15% in general practitioners’ practices. There was a substantial variation between disciplines and study groups, with relatively more consultations with general practitioners and relatively fewer consultations with paediatricians in the TR group and no consultations with general practitioners in the control group.

**Table 4:** Description of patient population

		Study group							
		Total (*n* = 127)		VR^a^ (*n* = 73)		TR^b^ (*n* = 14)		CG^c^ (*n* = 40)	
		N	%	N	%	N	%	N	%
Gender	Male	47	37.0	24	32.9	4	28.6	19	47.5
	Female	63	49.6	42	57.5	8	57.1	13	32.5
	Inter/Diverse	0	0.0	0	0.0	0	0.0	0	0.0
	Did not wish to answer	7	5.5	5	6.8	1	7.1	1	2.5
	Missing	10	7.9	2	2.7	1	7.1	7	17.5
Age	< 30	31	24.4	19	26.0	4	28.6	8	20.0
	30–39	31	24.4	21	28.8	2	14.3	8	20.0
	40–49	16	12.6	12	16.4	3	21.4	1	2.5
	> 50	6	4.7	4	5.5	2	14.3	0	0.0
	Missing	43	33.9	17	23.3	3	21.4	23	57.5
Highest school-leaving qualification	None	35	27.6	19	26.0	3	21.4	13	32.5
	Secondary school	41	32.3	29	39.7	2	14.3	10	25.0
	Technical school, high school or other	34	26.8	17	23.3	8	57.1	9	22.5
	Did not wish to answer	5	3.9	4	5.5	0	0.0	1	2.5
	Missing	12	9.4	4	5.5	1	7.1	7	17.5
Highest professional qualification	None	54	42.5	34	46.6	6	42.9	14	35.0
	Apprenticeship or other	12	9.4	9	12.3	1	7.1	2	5.0
	College, university degree or doctorate	20	15.8	9	12.3	6	42.9	5	12.5
	Did not wish to answer	8	6.3	5	6.8	0	0.0	3	7.5
	Missing	33	26.0	16	21.9	1	7.1	16	40.0
Language	Turkish	4	3.1	1	1.4	0	0.0	3	7.5
	Arabic	57	44.9	34	46.6	7	50.0	16	40.0
	Farsi	24	18.9	11	15.1	3	21.4	10	25.0
	Dari	15	11.8	10	13.7	3	21.4	2	5.0
	Kurdish	2	1.6	1	1.4	0	0.0	1	2.5
	Russian	9	7.1	4	5.5	0	0.0	5	12.5
	Tigrinya	16	12.6	12	16.4	1	7.1	3	7.5
	Missing	0	0.0	0	0.0	0	0.0	0	0.0
Specialty of physician consulted	General practitioner	19	15.0	8	11.0	11	78.6	0	0.0
	Obstetrician/ Gynaecologist	30	23.6	23	31.5	2	14.3	5	12.5
	Paediatrician	78	61.4	42	57.5	1	7.1	35	87.5
	Missing	0	0	0	0	0	0	0	0

^a^ = video remote interpreting, ^b^ = telephone remote interpreting, ^c^ = control group

### Questionnaire scores

A total of 93 (73.2%) PEI questionnaires, 91 (71.7%) PEF-FB-9 questionnaires and 98 (77.2%) PQC questionnaires were correctly completed and returned by the patients. Overall median values ranged from 87,50 (PEI) to 95,24 (PQC). The VR group scored highest in all three questionnaires. A detailed comparison of the questionnaires’ median values is displayed in Table 5.

**Table 5:** Median values and Interquartile Ranges (IQR) of PEI, PEF-FB-9, PQC, and outcome measures of the statistical tests conducted

Questionnaire	Descriptive measures	Total	VR	TR	CG	Hypothesis VR = TR = CG	TR-CG	TR-VR	CG-VR
PEI	N	93	54	13	26	*p* = 0.005^a^	*p* = 1.000^b^	*p* = .022^b^	p = .048^b^
	Median (IQR)	87.50 (75.00–100.00)	97.92 (79.17–100.00)	83.33 (75.00–85.42)	81.25 (61.46–100.00)				
PEF-FB-9	N	91	51	13	27	*p* = 0.022^a^	*p* = .601^b^	p = 1.000^b^	p = .018^b^
	Median (IQR)	94.44 (68.52–100.00)	96.30 (83.33–100.00)	92.59 (68.52–100.00)	72.22 (51.85–100.00)				
PQC Patients	N	98	55	13	30	p < 0.001^a^	*p* = .450^b^	*p* = .296^b^	p < .001^b^
	Median (IQR)	95.24 (85.71–100.00)	100.00 (90.48–100.00)	92.86 (80.95–100.00)	95.24 (85.71–100.00)				
PQC Physicians	N	178	111	14	53	p < 0.001^a^	p < .001^b^	p = 1.000^b^	*p* < .001^b^
	Median (IQR)	95.24 (76.19–100.00)	97.62 (92.86–100.00)	98.81 (94.64–100.00)	64.29 (54.76–76.19)				

^a^ Kruskal-Wallis test

^b^ Dunn-Bonferroni test

The Kruskal-Wallis tests showed significant differences for all three scores. Dunn-Bonferroni tests were performed to determine which groups showed significant differences. The scores between CG and VR (adjusted *p* = .048) and between TR and VR (adjusted *p* = .018) significantly differed for PEI. PEF-FB-9 showed significant differences between CG and VR (adjusted p = .018) and PQC significantly differed between CG and VR (adjusted *p* < .001). The outcome measures of the statistical tests performed are also displayed in Table 5.

### Subgroup analysis

A subgroup analysis was conducted comparing the outcomes of the control group to the VR group in paediatric care settings. The Mann-Whitney tests showed significant differences in all three occurring measures (*p* = 0.005 for PEI, *p* = 0.040 for PEF-FB-9, *p* < 0.001 for PQC).

### Physicians’ questionnaires

#### Sociodemographic data of the physicians

Six of the 13 physicians were male (46%). Nine physicians were between 41 and 60 years old (69%) and the same number had more than 20 years of professional experience. The majority of VR and TR groups were male physicians while the control group consisted only female physicians. Table 6 presents a detailed overview of the physicians’ sociodemographic data.

**Table 6:** Sociodemographic data of physicians

		Study group							
		Total		VR		TR		CG	
		N	%	N	%	N	%	N	%
Sex	Male	6	46.2%	3	60.0%	3	60.0%	0	0.0%
	Female	7	53.8%	2	40.0%	2	40.0%	3	100.0%
Age	≤40	1	7.7%	0	0.0%	1	20.0%	0	0.0%
	41–60	9	69.2%	5	100.0%	2	40.0%	2	66.7%
	> 60	3	23.1%	0	0.0%	2	40.0%	1	33.3%
Professional experience (years)	≤5	1	7.7%	0	0.0%	1	20.0%	0	0.0%
	6–10	1	7.7%	1	20.0%	0	0.0%	0	0.0%
	11–15	1	7.7%	0	0.0%	0	0.0%	1	33.3%
	16–20	1	7.7%	1	20.0%	0	0.0%	0	0.0%
	21–25	4	30.8%	2	40.0%	2	40.0%	0	0.0%
	> 25	5	38.5%	1	20.0%	2	40.0%	2	66.7%

### Questionnaire scores

Overall, 178 (88.1%) PQC questionnaires were returned by the physicians. 111 belonged to the VR group, 14 to the TR group and 53 to the control group. All 178 questionnaires returned were correctly completed. Mean values ranged from 64.65 for the control group to 96.26 for the TR group. A comparison of the mean values is displayed in Table 5.

The Kruskal-Wallis test showed significant differences for physicians’ PQC. Dunn-Bonferroni tests were performed to determine which of these groups exhibited significant differences. Major differences were found between CG and VR (adjusted *p* = .000) and CG and TR (adjusted p = .000). The results of the statistical tests performed are presented in Table 5.

## Discussion

Our exploratory pilot study highlights the discrepancy between the assumed high demand of professional interpreting solutions and the difficulties we experienced during the practice recruitment process. We were also surprised by the relatively low take-up rates. While interpreting costs were covered by the refugee first reception centres [38], the vague assessment of these costs presented a major obstacle to adequate healthcare provision outside these centres [27]. We had expected that offering a free, professional solution to the undeniable problem of a language barrier would lead to a high general interest in participating in this kind of study. However, this was markedly contrasted by a limited willingness to do so. Only 13 of the 593 medical practices contacted could be recruited; two fewer than we had originally aimed for. Moreover, interpreting tools were used far less often than we had expected. This discrepancy can be partly be attributed to the fact that we did not provide the medical practices with any incentives for participating apart from providing interpreting tools and taking part in a scientific project. Indeed, most medical practices treating language-discordant patients on a regular basis stated that they had no time to introduce such a tool into everyday practice or relied on other (informal) ways to bridge the language gaps. Although we can only speculate as to the exact reason for this, our findings suggest that many physicians either may not be confronted with language barriers or have instead found suitable solutions to overcome such obstacles. Meanwhile other physicians may not feel comfortable with an unknown third party entering the context of a medical intervention, or simply do not consider the use of an interpreter to be beneficial to the process. The fact that out of all the practices that were contacted during the recruitment process (593 in total), only the thirteen practices listed in Table 1 agreed to participate may have led to a selection bias among our study participants, meaning we included mainly healthcare professionals who were more strongly affected or confronted with language barriers. Despite all of this, we managed to recruit medical practices from all over the city of Hamburg and pertaining to all three primary care specialties (general medicine, obstetrics and gynaecology, paediatric care).

The small sample size of this study must however be considered a limitation. Due to the relatively small number of patients that were included, all statistical outcomes must be considered with caution. Different specialties (general medicine, obstetrics and gynecology, paediatric care) were also not equally allocated equally to all three study groups. Differences in outcome measures could possibly be attributed to a certain medical specialty rather than to the intervention applied. We tried addressing this matter by conducting a subgroup analysis comparing the 42 paediatric patients of the VR group with the 35 paediatric patients of the control group. The promising results of this subgroup analysis put this limitation into perspective and supported our findings that VR was rated as positive by both patients and doctors.

The commissioning of the interpreting tools themselves went smoothly. Within two weeks all medical practices had received their interpreting tools and were ready to start collecting data. Very few technical problems occurred throughout the study period. A total of 202 VR and TR calls were successfully conducted and nearly all physicians and patients rated the service provision very highly. More than 90% stated that they would benefit from further use of the tool in the future, showing a broad acceptance of remote interpreting tools among their users.

However, the tools were still much less relied on than we had expected. Other studies experienced the same problem [39] and identified several reasons for it [40].. The focus group broadly discussed these issues after the data collection period and the findings of this part of our study remain unpublished. The full implementation of the interpreting tools into everyday practice required a certain habituation process. Mottelson et al. also found that satisfaction with video interpreting increased with its usage [41]. It took six months to properly establish VR usage in a different study [42]. A broader reliance on remote interpreting services could only be a matter of time, especially in the context of the recent push towards digitalised communication precipitated by the COVID-19 pandemic. Feedback also mentioned that the questionnaires were too lengthy, which at times had prevented physicians from including a patient in the study. This should be remedied in future research projects. Almost 10 % of the total 202 calls were made in languages we had originally not included in the study. The geographical distribution of non-native German speakers over the city of Hamburg is dynamic and changes rapidly. This was already the case while planning and conducting our research, some medical practices ended up using languages that were not included in our study and others could not include as many patients as we had originally anticipated. The general medicine practice of the control group did not include a single patient for this reason. Consequently, the set of languages included in the study may need to be reconsidered and possibly adapted in future research projects. Furthermore, the languages needed may have to be assessed individually for every practice and every neighbourhood. This finding strikes us as one of the many advantages supporting the use of remote interpreting. Once such tools are implemented a broad variety of languages can become immediately available in any area.

Bearing in mind the general advantages of professional interpreters as listed in our introduction, we introduced professional remote interpreters as easily accessible professional interpreters to primary care practices all over Hamburg. Among the participating medical practices, physicians and patients of both remote interpreting groups were more satisfied with the communication than physicians and patients of the control group. This seems to be in line with broad sections of literature in this field, where the use of remote interpreting services generally result in high satisfaction rates [24, 43] and are more satisfactory than informal interpreters [44].

We achieved similar results could be found for the assessment of patient-centred medicine where patients scored higher in the intervention groups compared to the control group. This was particularly significant given the fact that patient-centred medicine did not only seem to lead to more patient satisfaction but also to better health outcomes [3, 4]. While VR scored very highly in terms of patient enablement, TR did not score as well. This finding might support the better acceptance of VR over TR in a study by Schulz et al. [45] but is contrary to the results of Jones et al. where PEI scores were high for TR but lower for VR [46]. However, all three studies only included a small number of patients for which the statistical results of the studies need to be treated with caution. In a systematic review comparing the satisfaction with TR and VR compared to in-person interpreting, Joseph et al. concluded that there was currently no evidence of a superior specific interpreting modality [25]. Further research is needed to determine with regards to which interpreting tool is in fact preferred. The tool best meets the demands of the patient or physician may also depend on individual circumstances. While scores for perceived quality of communication as well as the enablement of patient-centred medicine were high in both intervention groups and higher than the ones obtained in the control group, one has to bear in mind that we only conducted a small exploratory pilot study and the number of patients included remained low. For this reason, all questionnaire results need to be treated with caution. As the number of patients included was particularly low in the TR group, the results for this group may be difficult to interpret. The smaller TR sample size certainly represents a limitation of our study at this present time. We should however preemptively note some possible disadvantages of this interpreting tool compared to VR: The lack of non-verbal communications [18], bad audio quality, occupied hands [47] or the inability to demonstrate any kind of therapeutical motion for the patient to relate to (e.g. how to correctly apply an asthma spray, subcutaneous injections etc.) [46] are mentioned throughout literature. Further research eluding the scope of this study will hopefully determine whether the potential disadvantages of TR result in lower quality of medical consultations.

When setting up head-to-head trials in different countries it should be kept in mind that researchers abroad may face different conditions. Questions, such as the ones concerning, amongst other, digital infrastructure, desired languages, existing ethnic groups, financing interpreting in medical consultations and other legal aspects must to be taken into account. Most of these questions also have to be considered throughout the course of implementing remote interpreting into primary health care. As medical practices are not scientifically driven, financial aspects, in particular, may be important to consider when implementing a solution for daily routine. The costs incurred by interpreting in medical settings seem to present a key challenge when implementing remote interpreting tools and services.

### Implications

Based on the feedback we received from medical professionals not wanting to participate, we believe that a certain lack of awareness regarding both the adverse effects of informal interpreting and the benefits of remote interpreting explains our difficulties in the recruitment process. This lack of awareness or unwillingness to explore the issue of language barriers for the reasons listed in the discussion need to be overcome. The demand for professional interpreting is obvious, with more than 90% of both patients as well as physicians stating that they would benefit from continued utilisation of the interpreting tool in the future. Future studies may have to offer incentives to include more medical practices, in order to investigate the implementation of professional remote interpreting services within primary care settings more thoroughly. While the provision of professional interpreting services appears to be very good at refugees’ first reception centres, they remain widely insufficient in the primary care sector. This discrepancy is, at least to some degree, rooted in the unclear cost assessment of such services in the primary care sector. For this reason, we suggest providing political solutions that unambiguously clarifies the costs assessment for interpreting services and straightforward responsibilities regarding the provision of professional interpreting in medical settings. This can be achieved by considering interpreting as an essential part of medical care and therefore including it on the list of medical services covered by health insurance. Moreover, future research needs to deliver more information on the implementation of remote interpreting into primary care settings with a focus on comparing different interpreting modalities. Finally, solutions must be found to guarantee the continued standard of medical care at medical practices that were able to rely on remote interpreting services during the period of the study but which are now, once again, left without these tools.

## Conclusion

To the best of our knowledge, this is the first study in the German language area comparing telephone remote interpreting to video remote interpreting with a control group in primary care settings. We found that it was feasible to implement remote interpreting services into everyday practice in primary care settings. The recruitment of medical practices represented an obstacle because it was a scientific project, but the technical implementation went smoothly and the interpreting tools could be broadly relied upon by their users. The results regarding perceived quality of communication and the enablement of patient-centred medicine appear to be promising and are worthy of further research. The focus of such further research should be on the implementation of professional remote interpreting services into primary care settings and a comparison of different interpreting modalities with larger study groups.

## Supplementary Information

**Additional file 1. **Presentation of the languages offered by *SAVD Videodolmetschen GmbH.*

**Additional file 2.** Acceptance of interpreting tools.

## Data Availability

The datasets supporting the conclusions of this study are included within the article and its additional files. The questionnaires forming the basis of these datasets as well as the pilot trial protocol can be found in the archive of the Department of General Practice / Primary Care, University Medical Center Hamburg-Eppendorf, Hamburg, Germany.

## Abbreviations

LEP: Limited English proficiency
VR: Video remote interpreting
TR: Telephone remote interpreting
CG: Control group
PEI: Patient Enablement Index
PEF-FB-9: Fragebogen zur partizipativen Entscheidungsfindung
SDM-Q-9: Decision-Making Questionnaire
