# Supplementary material for: Remote interpreting in primary care settings: a feasibility trial in Germany
Source: BMC Health Serv Res. 2022 Jan 24;22:99. doi: 10.1186/s12913-021-07372-6 (PMC8785004; doi:10.1186/s12913-021-07372-6)
Supplement: Supplementary file 1 — Additional file 1. Presentation of the languages offered by SAVD Videodolmetschen GmbH. [file 12913_2021_7372_MOESM1_ESM.docx]

## Appendix 1 – Presentation of the languages offered by *SAVD Videodolmetschen GmbH*

**Table of languages**
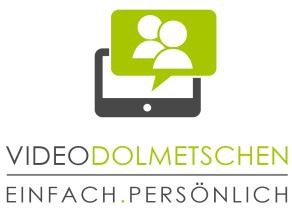


| **Language** | **Native name** |  |
| --- | --- | --- |
| German | Deutsch | Welche Sprache Sprechen Sie? |
| Albanian | Shqip | Cilën gjuhë e flisni? |
| Amharic | ኣማርኛ | የትኛውን ቋንቋ ይናገራሉ ? |
| Arabic | عربيي | أي لغة تتكلم؟ |
| Armenian | ՀԱՅԵՐԵՆ | Ի՞նչ լեզվով եք խոսում: |
| Azerbaijani | Azərbaycanca | Hansı dildə danışırsız? |
| Bengali | বাাংলা | আপনি ক াি ভাষা বললি ? |
| Bosnian | Bosanski | Koji jezik govorite? |
| Bulgarian | Български | Какъв език говорите? |
| Chinese - Mandarin | 汉语 - 普通话 | 您说哪种语言？ |
| Croatian | Hrvatski | Koji jezik govorite? |
| Czech | čeština | Jakým jazykem mluvíte? |
| Dari | دری | به کدام زبان صحبت ميکنيد؟ |
| English | English | What language do you speak? |
| Estonian | eesti keel | Mis keelt te räägite? |
| Farsi | فارسی | زبان شما چيست؟ |
| Filipino | Filipino | Ano ang inyong wika? |
| French | Français | Quelle langue parlez-vous? |
| Georgian | ქართული | რა ენაზე მეტყველებთ? |
| Greek | Ελληνικά | Τι γλώσσα μιλάτε; |
| Hebrew | עברית | איזה שפה אתה מדבר? |
| Hindi | ह दिंी | आप कौन सी भाषा बोलते ैं |
| Hindi |  | Aap kaun see bhaasha bolate hain? |
| Hungarian | Magyarul | Milyen nyelven beszél? |
| Igbo | Asụsụ Igbo | Kedu asusu i na-asu? |
| Indonesian | Bahasa Indonesia | Bahansa yang Anda berbicara? |
| Italian | Italiano | Quale lingua parla? |
| Korean | 한국어 | 어떤 언어를 사용하십니까? |
| Kurdish - Kurmanji | Kurdi – Kurmanci | Hun ki jan ziman i di axifin? |
| Kurdish - Sorani | كوردی سۆرانی | زمانی ئێوە چێ؟ |
| Latvian | Latviešu valoda latviski | Kādā valodā Jūs runājat? |

**Language Native name**
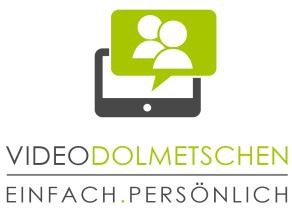


| Lithuanian | lietuvių kalba Kokia kalba Jūs kalbate? | |
| --- | --- | --- |
| Malinke | Maninka kan Alube kan juma foola? | |
| Mongolian | МОНГОЛ ТА ЯМАР ХЭЛЭЭР ЯРЬДАГ ВЭ? | |
| Nepali | नेपाली तपाईँ कुन भाषा बोल्न ु ुन्छ? | |
| Oromo | afaan oromoo Afaan isa kam dubbattu? | |
| Pashto | تاسی په کومه ژبه خبری کوۍ ؟ (پشتو) | |
| Polish | polski Jakim językiem mówisz? | |
| Portuguese | Português Que língua fala? | |
| Punjabi (Indian) | ਪੰਜਾਬੀ ਤੁਸੀਂ ਕਿਹੜੀ ਭਾਸਾ ਬੋਲਦੇ ਹੋ^?^ | |
| Punjabi (Pakistani) | تُسی کيہڑی زُبان بولدے ہو؟ پنجابی | |
| Rumanian | Română Ce limbă vorbiți? | |
| Russian | русский язык На каком языке Вы говорите? | |
| Serbian | Srpski/ Српски Koji jezik govorite?/ Који језик говорите? | |
| Slowakian | slovenčina | Akou rečou hovoríte? |
| Slovenian | Slovensko | Kateri jezik govorite? |
| Somali | Af - Soomaali | Luqadeed ku hadashaa? |
| Spanish | Español | ¿Qué idioma habla usted? |
| Suaheli | Kiswahili | Unazungumza lugha gani? |
| Susu | Soso khui | Wo khui munndun falama? |
| Tamil | தமிழ் | நீங்கள் என்ன மமொழி பேசுவர்ீகள்? |
| Tigrinya | ትግርኛ | እንታይ ቋንቋ ትዛረቡ? |
| Turkish | Türkçe | Hangi dili konuşuyorsunuz? |
| Twi | Twi | Kasa ben, na wo ka? |
| Ukranian | українська мова | Якою мовою Ви розмовляєте? |
| Urdu | ارُدو | آپ کونسی زُبان بولتے ہيں؟ |
| Vietnamese | tiếng Việt | Ngài nói được ngôn ngữ nào? |
| Zarma |  | Ciine fonoo ni ga salan? |
