# Supplementary material for: Remote interpreting in primary care settings: a feasibility trial in Germany
Source: BMC Health Serv Res. 2022 Jan 24;22:99. doi: 10.1186/s12913-021-07372-6 (PMC8785004; doi:10.1186/s12913-021-07372-6)
Supplement: Supplementary file 2 — Additional file 2. Acceptance of interpreting tools. [file 12913_2021_7372_MOESM2_ESM.docx]

| Appendix 2 – Acceptance of interpreting tools **Table 7** –Patients’ acceptance of interpreting tools | | | | | | | |
| --- | --- | --- | --- | --- | --- | --- | --- |
|  | | Study group | | | | | |
|  |  | Total | | VR | | TR | |
|  |  | N | % | N | % | N | % |
| Tool facilitated communication with the physician | completely disagree | 0 | 0.0% | 0 | 0.0% | 0 | 0.0% |
|  | mostly disagree | 1 | 1.3% | 1 | 1.6% | 0 | 0.0% |
|  | somewhat disagree | 0 | 0.0% | 0 | 0.0% | 0 | 0.0% |
|  | somewhat agree | 3 | 3.9% | 2 | 3.2% | 1 | 7.1% |
|  | mostly agree | 15 | 19.7% | 12 | 19.4% | 3 | 21.4% |
|  | completely agree | 57 | 75.0% | 47 | 75.8% | 10 | 71.4% |
| Tool helped me to better present my issue | completely disagree | 0 | 0.0% | 0 | 0.0% | 0 | 0.0% |
|  | mostly disagree | 0 | 0.0% | 0 | 0.0% | 0 | 0.0% |
|  | somewhat disagree | 2 | 2.7% | 2 | 3.4% | 0 | 0.0% |
|  | somewhat agree | 3 | 4.1% | 2 | 3.4% | 1 | 7.1% |
|  | mostly agree | 11 | 15.1% | 9 | 15.3% | 2 | 14.3% |
|  | completely agree | 57 | 78.1% | 46 | 78.0% | 11 | 78.6% |
| Tool helped me to better understand the physician's questions and explanations | completely disagree | 1 | 1.4% | 1 | 1.7% | 0 | 0.0% |
|  | mostly disagree | 0 | 0.0% | 0 | 0.0% | 0 | 0.0% |
|  | somewhat disagree | 0 | 0.0% | 0 | 0.0% | 0 | 0.0% |
|  | somewhat agree | 5 | 6.9% | 3 | 5.2% | 2 | 14.3% |
|  | mostly agree | 11 | 15.3% | 9 | 15.5% | 2 | 14.3% |
|  | completely agree | 55 | 76.4% | 45 | 77.6% | 10 | 71.4% |
| User-friendly tool | completely disagree | 0 | 0.0% | 0 | 0.0% | 0 | 0.0% |
|  | mostly disagree | 0 | 0.0% | 0 | 0.0% | 0 | 0.0% |
|  | somewhat disagree | 0 | 0.0% | 0 | 0.0% | 0 | 0.0% |
|  | somewhat agree | 3 | 4.1% | 2 | 3.4% | 1 | 7.1% |
|  | mostly agree | 11 | 15.1% | 8 | 13.6% | 3 | 21.4% |
|  | completely agree | 59 | 80.8% | 49 | 83.1% | 10 | 71.4% |
| I would benefit from further use of the tool in the future | completely disagree | 0 | 0.0% | 0 | 0.0% | 0 | 0.0% |
|  | mostly disagree | 0 | 0.0% | 0 | 0.0% | 0 | 0.0% |
|  | somewhat disagree | 0 | 0.0% | 0 | 0.0% | 0 | 0.0% |
|  | somewhat agree | 6 | 8.0% | 5 | 8.2% | 1 | 7.1% |
|  | mostly agree | 12 | 16.0% | 9 | 14.8% | 3 | 21.4% |
|  | completely agree | 57 | 76.0% | 47 | 77.0% | 10 | 71.4% |

| Table 8 –Physicians’ acceptance of interpreting tools | | | | | | | |
| --- | --- | --- | --- | --- | --- | --- | --- |
|  | | Study group | | | | | |
|  |  | Total | | VR | | TR | |
|  |  | N | % | N | % | N | % |
| Tool facilitated communication with the patient | completely disagree | 1 | 0.8% | 1 | 0.9% | 0 | 0.0% |
|  | mostly disagree | 0 | 0.0% | 0 | 0.0% | 0 | 0.0% |
|  | somewhat disagree | 0 | 0.0% | 0 | 0.0% | 0 | 0.0% |
|  | somewhat agree | 0 | 0.0% | 0 | 0.0% | 0 | 0.0% |
|  | mostly agree | 4 | 3.2% | 2 | 1.8% | 2 | 14.3% |
|  | completely agree | 120 | 96.0% | 108 | 97.3% | 12 | 85.7% |
| Tool helped me to better understand the patient | completely disagree | 1 | 0.8% | 1 | 0.9% | 0 | 0.0% |
|  | mostly disagree | 0 | 0.0% | 0 | 0.0% | 0 | 0.0% |
|  | somewhat disagree | 0 | 0.0% | 0 | 0.0% | 0 | 0.0% |
|  | somewhat agree | 0 | 0.0% | 0 | 0.0% | 0 | 0.0% |
|  | mostly agree | 6 | 4.8% | 2 | 1.8% | 4 | 28.6% |
|  | completely agree | 118 | 94.4% | 108 | 97.3% | 10 | 71.4% |
| Tool helped the patient to better understand me | completely disagree | 1 | 0.8% | 1 | 0.9% | 0 | 0.0% |
|  | mostly disagree | 0 | 0.0% | 0 | 0.0% | 0 | 0.0% |
|  | somewhat disagree | 0 | 0.0% | 0 | 0.0% | 0 | 0.0% |
|  | somewhat agree | 0 | 0.0% | 0 | 0.0% | 0 | 0.0% |
|  | mostly agree | 9 | 7.2% | 4 | 3.6% | 5 | 35.7% |
|  | completely agree | 115 | 92.0% | 106 | 95.5% | 9 | 64.3% |
| User-friendly tool | completely disagree | 1 | 0.8% | 1 | 0.9% | 0 | 0.0% |
|  | mostly disagree | 1 | 0.8% | 1 | 0.9% | 0 | 0.0% |
|  | somewhat disagree | 1 | 0.8% | 1 | 0.9% | 0 | 0.0% |
|  | somewhat agree | 1 | 0.8% | 1 | 0.9% | 0 | 0.0% |
|  | mostly agree | 17 | 13.6% | 14 | 12.6% | 3 | 21.4% |
|  | completely agree | 104 | 83.2% | 93 | 83.8% | 11 | 78.6% |
| I would benefit from further use of the tool in future consultations with this patient | completely disagree | 0 | 0.0% | 0 | 0.0% | 0 | 0.0% |
|  | mostly disagree | 0 | 0.0% | 0 | 0.0% | 0 | 0.0% |
|  | somewhat disagree | 0 | 0.0% | 0 | 0.0% | 0 | 0.0% |
|  | somewhat agree | 0 | 0.0% | 0 | 0.0% | 0 | 0.0% |
|  | mostly agree | 2 | 1.6% | 1 | 0.9% | 1 | 7.1% |
|  | completely agree | 123 | 98.4% | 110 | 99.1% | 13 | 92.9% |
